# Hydroxycarboxylic acid receptor 2 (GPR109A) and retinopathies: pathways and prospects

**DOI:** 10.3389/fmed.2026.1776648

**Published:** 2026-03-11

**Authors:** John Lester, Ronny Amamoo, Menaka C. Thounaojam, Pamela M. Martin, Ravirajsinh N. Jadeja

**Affiliations:** 1 Department of Biochemistry and Molecular Biology, Medical College of Georgia at Augusta University, Augusta, GA, United States; 2 Department of Biomedical Sciences, School of Graduate Studies, Meharry Medical College, Nashville, TN, United States

**Keywords:** beta-hydroxy butyrate, butyrate, G-protein coupled receptor, niacin, retinal diseases, retinopathy

## Abstract

GPR109A, also known as the hydroxycarboxylic acid receptor 2 (HCAR2), is a G protein–coupled receptor with emerging significance in ocular health. Although considerable attention has focused on its role in the diabetic retina, growing evidence suggests that GPR109A may also play an important role in other retinal pathologies, including hypertensive retinopathy (HR) and retinopathy of prematurity (ROP), where inflammation, oxidative stress, and vascular instability similarly drive disease progression. Expressed in key retinal cell types, including retinal pigment epithelial cells, endothelial cells, and microglia, GPR109A mediates anti-inflammatory, antioxidant, and barrier-protective effects through activation by endogenous ligands such as niacin, β-hydroxybutyrate (BHB), and butyrate, as well as synthetic agonists, including monomethyl fumarate (MMF) and L-2-oxothiazolidine-4-carboxylic acid (OTC). This review highlights the broader therapeutic potential of targeting GPR109A across multiple retinal diseases, emphasizing early-stage intervention and opportunities for non-invasive treatment strategies. We also discuss the efficacy and limitations of GPR109A agonists, including those that activate both GPR109A-dependent and receptor-independent pathways, and explore the potential of biased agonism to reduce systemic side effects such as cutaneous flushing. While preclinical data are compelling, further studies are needed to optimize delivery methods, validate efficacy in clinical settings, and overcome translational challenges. Overall, GPR109A represents a promising frontier in the development of preventive therapies for vision-threatening retinal disorders, extending well beyond diabetic retinopathy to conditions such as HR and ROP.

## 1 Introduction

Retinopathy encompasses a spectrum of retinal vascular diseases, including hypertensive retinopathy (HR), retinopathy of prematurity (ROP), and diabetic retinopathy (DR) (1). While the underlying causes of these conditions are distinct hypertension in HR, hyperoxia in ROP, and hyperglycemia in DR, their pathophysiological mechanisms converge on a common outcome: damage to the retinal microvasculature (1). This damage is exacerbated by oxidative stress and pro-inflammatory signaling, which contribute to retinal ischemia and the release of pro-angiogenic factors such as vascular endothelial growth factor (VEGF). The resulting pathological neovascularization drives disease progression and ultimately leads to vision loss (1).

HR is a complication of systemic hypertension, characterized by structural and functional changes within the retinal vasculature. It is a product of end-organ damage within the eye, and evidence indicates that the incidence of HR correlates with the severity and chronicity of hypertension (2). This allows the retinal vasculature to serve as a window into systemic vascular health. The prevalence of this disease varies greatly amongst different populations, but ranges from 2% to 17% in non-diabetic patients. Clinically, it can be diagnosed via the presence of arteriovenous nicking, arteriolar narrowing, or hemorrhages and microaneurysms in more advanced cases (3). Retinal vessels use local autoregulatory mechanisms to control pressure and blood flow, as they lack sympathetic nervous input; thus, elevated blood pressure is transmitted directly to the vasculature. This triggers an initial compensatory vasoconstriction, but once compensatory mechanisms fail, the vessel wall becomes sclerotic. Eventually, chronic hypertension disrupts the blood-retinal barrier, which allows for the hemorrhages and microaneurysms seen in moderate to advanced HR. These changes result in the exudate formation and ischemia seen clinically (3). Treatment of HR centers on the reduction of systemic blood pressure with common anti-hypertensive agents such as ACE inhibitors, angiotensin receptor blockers, etc (4). However, little research has been done on targeting the pathological neovascularization seen in this disease. There are sparse case reports noting the efficacy of intraocular injections of anti-VEGF antibodies; however, these are invasive treatments and applicable only to severe disease (5, 6).

ROP is unique to pre-term and low birth weight infants, and the leading cause of irreversible vision loss in childhood (7). It arises when normal retinal vascularization is disrupted and leads to premature infants at high risk of pathological neovascularization and potential retinal detachment. Each year, approximately 20,000 infants are diagnosed with severe visual impairment or blindness, with another 12,000 experiencing irreversible visual loss (8). Clinically, ROP can be a difficult diagnosis, as pre-term and or low birth weight infants may have a myriad of health concerns. In infants with ROP, a dilated fundus exam may reveal characteristic retinal changes. These range from a demarcation line separating vascularized and avascular retina to total retinal detachment (9). The pathophysiology of ROP occurs in two phases. The relative hypoxia *in utero* promotes structured vascularization within the retina. Once born, premature infants are exposed to relative hyperoxia, especially if they require supplemental oxygenation due to an underdeveloped respiratory system. This inhibits VEGF expression, halting retinal neovascularization. Phase 1 is known as the vaso-obliterative phase (10, 11). As normal neovascularization is halted, the peripheral retina is left largely avascular and becomes hypoxic. In response, there is an overproduction of VEGF and other angiogenic growth factors, leading to pathological neovascularization. These abnormal vessels are fragile and grow in an unstructured way, leading to the retinal hemorrhage and fibrovascular proliferation seen in phase 2 of ROP (9, 12). Current management of ROP is similar to other forms of retinopathy and includes laser photocoagulation and anti-VEGF injections. To reduce VEGF production, lasers are used to burn avascular or ischemic areas of the retina. Laser therapy is known to carry a significant risk of refractive issues such as myopia (13, 14). Evidence for anti-VEGF injections in the treatment of ROP is scarce, with studies showing it fails to reduce the risk of retinal detachment when used as monotherapy (15). Current treatment shortcomings necessitate a non-invasive therapy with a more favorable side effect profile.

DR is the leading cause of permanent vision loss and blindness among adults in industrialized countries (16, 17). As of 2021, roughly 11.6% of the US population had diabetes (7). Among diabetic individuals, nearly all patients with type 1 diabetes and more than 60% of those with type 2 diabetes develop DR within the first 20 years of disease onset. Diagnostically, DR presents a challenge because symptom onset often occurs after significant retinal damage has already taken place (18). Hyperglycemia is a primary driver of DR pathogenesis, initiating a cascade of metabolic and inflammatory changes that compromise retinal vascular integrity. Although maintaining glycemic control can slow disease progression, it does not halt it entirely (19). Current treatments for DR, such as laser ablation, anti-VEGF injections, and vitrectomy, are invasive and only applicable in advanced stages when major microvascular changes have occurred. These interventions are associated with adverse outcomes, particularly anti-VEGF injections, which can lead to vitreous hemorrhage and further vision deterioration (20). Thus, there is a pressing need for novel DR treatments focused on prevention rather than end-stage intervention. Inflammation has long been recognized as a major contributor to the onset and progression of DR (19, 21–23). Chronic inflammatory signaling disrupts the blood-retina barrier, promotes leukocyte infiltration, and exacerbates tissue damage. Oxidative stress, driven by reactive oxygen species (ROS), further impairs cellular function and accelerates disease progression. These interlinked processes underscore the need for therapeutic strategies that target early molecular events rather than late-stage structural damage. Therapies that can act earlier, prior to the clinical onset of DR symptoms, may have the potential to inhibit or delay progression to late-stage retinal disease. Such interventions could preserve vision and improve the quality of life for millions of people worldwide.

In this context, GPR109A, a G-protein-coupled receptor expressed in the retina, has emerged as a promising therapeutic target. Initially identified in adipocytes as a receptor for niacin, GPR109A is now known to be expressed in retinal pigment epithelial cells, retinal endothelial cells, and microglia. Activation of GPR109A by endogenous ligands such as β-hydroxybutyrate (BHB) and butyrate, or by pharmacologic agents such as niacin, has been shown to exert anti-inflammatory, antioxidant, and barrier-protective effects. These properties position GPR109A as a compelling candidate for non-invasive, early-stage treatment strategies in retinopathies and other ocular diseases. Throughout this review, we discuss the experimental evidence, both within and outside the retina, that supports this therapeutic potential.

## 2 Discovery and biology of GPR109A

In 2003, three groups independently discovered hydroxycarboxylic acid receptor 2 (HCAR2), also known as GPR109A (24). It was identified as a high-affinity receptor for niacin at the time of its discovery and hence also named niacin receptor 1 (NIACR1) (24). Since the 1950s, niacin has been used to treat hyperlipidemia, specifically by increasing high-density lipoproteins (HDL) and decreasing low-density lipoproteins (LDL). Nearly 70 years later. Even with the introduction of new agents such as statins (HMG-CoA reductase inhibitors) and bile acid resins, niacin is still used therapeutically, as the newer agents lower LDL levels with minimal effect on HDL levels (24–27). In addition to its efficacy in improving lipid/apolipoprotein profiles, niacin has anti-inflammatory effects that have been shown to decrease vascular inflammation. This subsequently reduces the risk of atherosclerosis and thrombosis, significantly lowering the risk of thrombotic events such as stroke and myocardial infarction. Studies have shown that niacin’s anti-inflammatory effects on the vasculature and endothelial cells stem directly from its activation of GPR109A (28).

After discovering GPR109A and its interactions with niacin, further studies identified other agonists, such as the endogenous ligands BHB and butyrate (29, 30). Another finding from these studies was that GPR109A expression was identified outside adipocytes, in various cell and tissue types. This implied that GPR109A served a physiological role apart from its effects on cholesterol metabolism when activated by niacin. For example, GPR109A activation in immune cells was found to suppress pro-inflammatory cytokines, inducing a strong anti-inflammatory response (31). These findings were significant, as immune cells such as macrophages are integral to the progression of atherosclerotic lesions and cardiovascular disease. Receptor activation in these cells elicited antioxidant and anti-inflammatory responses like those observed in immune cells (32, 33). The effects of GPR109A’s activation by the endogenous agonists BHB and butyrate revealed an additional physiological role for the receptor in monitoring nutrient levels and regulating energy homeostasis (34, 35).

## 3 GPR109A signaling mechanisms

### 3.1 GPCR structure and G-protein coupling (Gi/o)

**FIGURE 1:**
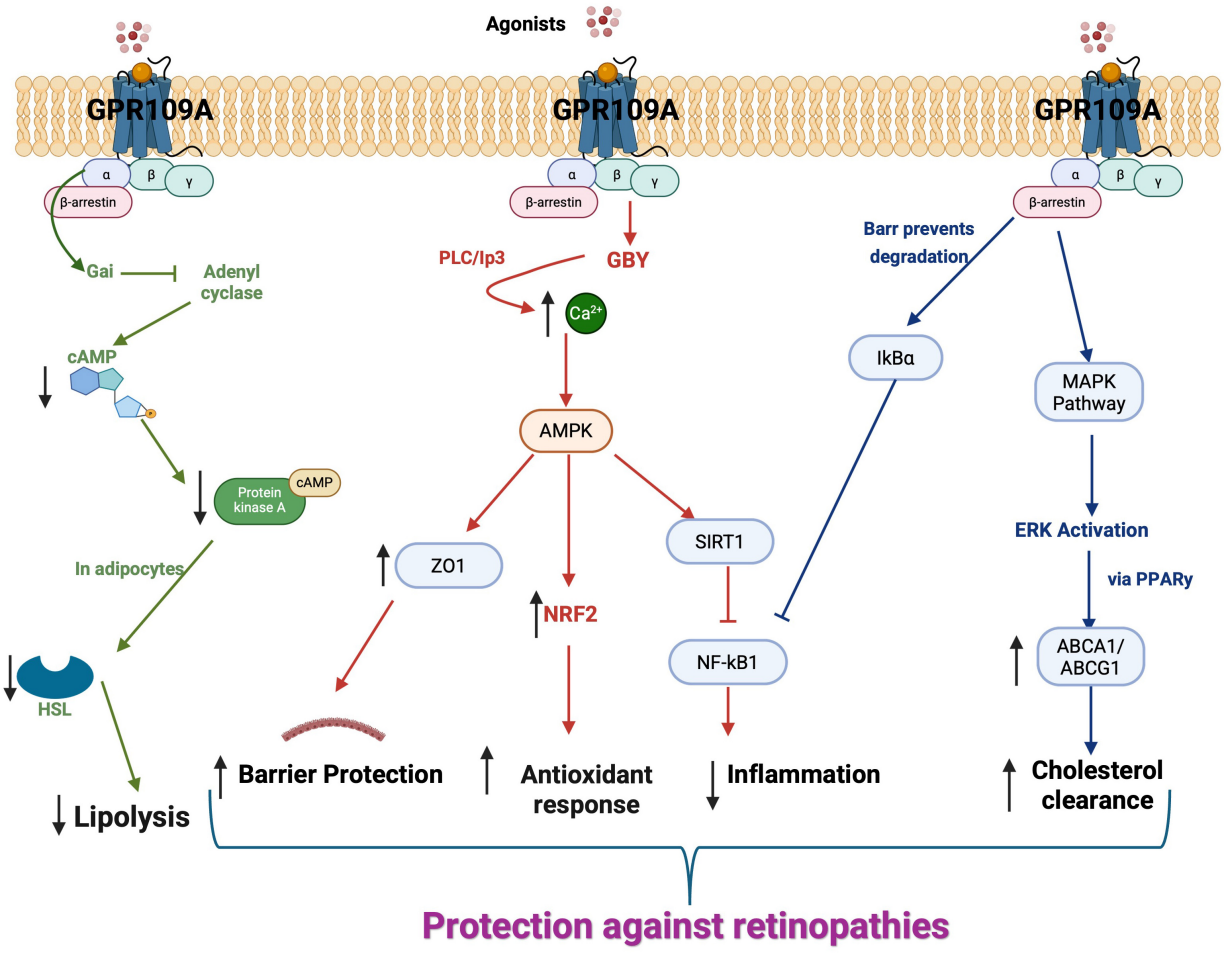
GPR109A signaling mechanism. The GPR109A receptor mediates diverse biological effects through distinct signaling pathways. Upon activation, GPR109A inhibits lipolysis in adipocytes via Gαi-dependent suppression of adenylyl cyclase, leading to reduced cAMP and downstream PKA and HSL activity. In RPE cells, GPR109A activation triggers Ca^2+^ release and AMPK signaling, enhancing barrier integrity (via ZO1), promoting antioxidation (via NRF2), and suppressing inflammation (via SIRT1-mediated NF-κB1 inhibition). Additionally, GPR109A stabilizes IκBα through β-arrestin interaction, inhibiting NF-κB signaling and activating the MAPK/ERK pathway via PPARγ, which upregulates ABCA1/ABCG1 transporters to promote HDL synthesis. GPR109A-mediated barrier protection, increased antioxidant response, reduced inflammation and increases cholesterol clearance provides protection against various retinopathies. Created with BioRender.com.

GPR109A belongs to the G-protein coupled receptor (GPCR) superfamily, characterized by a conserved seven-transmembrane helical architecture. In simpler terms, activation of GPR109A triggers a well-established GPCR mechanism in which the receptor acts as a molecular switch, initiating downstream signaling through G-proteins. Upon agonist binding, GPCRs act as guanine nucleotide exchange factors (GEFs), promoting the release of GDP from the Gα subunit of the heterotrimeric G-protein complex (36, 37). Gα then binds GTP, triggering conformational changes and dissociation from the Gβγ subunits (38). GPR109A specifically couples to the inhibitory Gαi/o subunit, leading to downstream suppression of adenylate cyclase activity (27) (Figure 1).

### 3.2 Downstream effects

Classically, activation of GPR109A inhibits adenylate cyclase, leading to reduced intracellular cAMP levels and, in turn, suppression of protein kinase A (PKA) activity. In adipocytes, this leads to decreased hormone-sensitive lipase activity and reduced triacylglyceride hydrolysis (26, 39). This antilipolytic effect is enhanced pharmacologically by niacin and other agonists. Beyond this primary pathway, GPR109A can activate several additional signaling routes that contribute to broader metabolic and inflammatory regulation. The downstream events described below outline how GPR109A activation influences key cellular processes, including inflammation, oxidative stress, and barrier protection.

#### 3.2.1 Anti-inflammatory pathways

GPR109A signaling extends beyond cAMP suppression (Figure 1). Activation of the Gi/o pathway has been shown to stimulate extracellular signal-regulated kinase (ERK) via β-arrestin scaffolding (40). ERK activation promotes prostaglandin D2 (PGD2) synthesis, which activates peroxisome proliferator-activated receptor gamma (PPAR-γ), a transcription factor involved in lipid metabolism (41). GPR109A undergoes agonist-induced internalization via GPCR kinase (GRK) and β-arrestin-dependent mechanisms (42, 43). β-arrestins mediate receptor desensitization and serve as scaffolds for signaling cascades, including the ERK and c-Jun N-terminal kinase 3 (JNK3) pathways (44–46). β-arrestin 1 has been shown to translocate to the nucleus, recruit histone acetyltransferase p300, and enhance histone H4 acetylation, thereby regulating gene transcription (47). It also interacts with NF-kappa-B inhibitor (IκBα) to inhibit NF-κB (NF-kappa-B) activation (48) and subsequently inflammation. Significantly, β-arrestin 1 mediates the cutaneous flushing side effect of niacin by activating cytosolic phospholipase A2 and releasing PGD2 in Langerhans cells and keratinocytes (40). This phenomenon highlights the potential for biased agonism, in which selective ligands can activate beneficial pathways while avoiding adverse effects.

#### 3.2.2 Antioxidant and metabolic stress-response pathways

Another important aspect of GPR109A signaling involves pathways that regulate cellular energy status and oxidative stress responses, as summarized below. GPR109A also modulates intracellular calcium via phospholipase C activation, leading to phosphorylation of AMP-activated protein kinase (AMPK) (49, 50) (Figure 1). AMPK activation enhances nicotinamide adenine dinucleotide (NAD^+^) levels and activates sirtuin 1 (SIRT1), thereby suppressing NF-κB signaling (51). Additionally, GPR109A/AMPK signaling promotes nuclear import of nuclear factor erythroid 2-related factor 2 (NRF2), a key regulator of antioxidant responses and autophagy (52). These antioxidant mechanisms highlight GPR109A as a potential therapeutic target for retinal diseases characterized by oxidative stress, including AMD and ROP.

#### 3.2.3 Barrier-protective pathways

GPR109A/AMPK signaling also facilitates tight junction formation by promoting zona occludin 1 (ZO-1) localization to the plasma membrane (53–58). By strengthening tight junction architecture, GPR109A supports blood–retina barrier integrity, which is critical for preventing vascular leakage in DR, HR, and early-stage ROP.

Together, these interconnected signaling pathways illustrate how GPR109A exerts diverse biological effects across multiple tissues, which helps contextualize its relevance to retinal disease.

## 4 GPR109A in systemic diseases relevant to ocular pathology

### 4.1 Vascular protection

GPR109A activation has been shown to exert protective effects on the cardiovascular system (59). Studies using LDL-receptor knockout mice demonstrated that niacin decreased the progression of atherosclerosis without altering LDL or HDL levels. This was accompanied by reduced expression of cellular adhesion molecules (CAMs) in atherosclerotic vessels (60). In humans, a randomized controlled trial revealed that 12 months of niacin supplementation significantly reduced carotid wall area, a known risk marker for coronary heart disease and stroke (61).

The anti-atherosclerotic effects of niacin are attributed to its ability to inhibit monocyte chemoattractant protein-1 (MCP-1). In adipocytes treated with tumor necrosis factor-alpha (TNF-α) and niacin, MCP-1 expression was significantly reduced compared to TNF-α alone (62). Niacin also upregulates adiponectin, an anti-inflammatory peptide hormone, via GPR109A activation. In human monocytes, niacin reduces TNF-α, MCP-1, and interleukin-6 (IL-6) expression and secretion by inhibiting toll-like receptors TLR4 and TLR2. These effects were abolished when GPR109A was knocked down using siRNA, confirming the receptor’s role (62).

Independent of niacin, butyrate, a short-chain fatty acid and endogenous GPR109A agonist, also reduces MCP-1 expression in macrophages and dendritic cells. Butyrate promotes IL-10 secretion and reduces vascular cell adhesion molecule-1 (VCAM-1) release, which is critical for macrophage recruitment and trans-endothelial migration (63, 64). These findings suggest that GPR109A activation can modulate early vascular inflammation and immune cell recruitment in various retinal diseases.

### 4.2 Oxidative stress

Oxidative stress, driven by ROS and LDL oxidation, is a key contributor to atherosclerosis. Ganji et al. (32) showed that niacin significantly reduced ROS production and LDL oxidation in human aortic endothelial cells exposed to angiotensin II. Niacin also decreased TNF-α-induced MCP-1 and VCAM-1 expression, highlighting its antioxidant and anti-inflammatory effects (32). Niacin’s ability to inhibit ROS and prevent LDL oxidation is central to its vascular protective effects. These mechanisms reduce endothelial dysfunction and the formation of fatty streaks, which are early indicators of atherosclerosis (65). Butyrate has also been shown to activate NRF2 via GPR109A. In bovine mammary epithelial cells exposed to hydrogen peroxide, butyrate treatment led to NRF2 accumulation and upregulation of antioxidant enzymes. Knockdown of GPR109A significantly reduced NRF2 levels and cell survival, confirming the receptor’s role in mitigating oxidative stress (66, 67). Since oxidative stress is a key component of various retinal diseases, GPR109A agonists could be used to reduce oxidative stress in ocular tissue.

### 4.3 Diabetes

GPR109A is expressed in podocytes, suggesting a role in renal protection. In a doxorubicin-induced nephropathy study, butyrate treatment improved proteinuria and reduced glomerulosclerosis in wild-type mice. These effects were absent in GPR109A knockout mice, indicating that podocyte protection is mediated by GPR109A activation (68). Butyrate increased podocyte-related proteins and regulated genes essential for podocyte function. This renoprotective effect was observed in both *in vivo* and *in vitro* models, reinforcing the importance of GPR109A in maintaining renal integrity under stress conditions. In renal tubular cells and podocytes exposed to hyperglycemic conditions, butyrate reduced the levels of inflammatory cytokines and fibrosis. Diabetic mice fed high-fiber diets showed increased butyrate production and reduced risk of diabetic nephropathy. These effects were abrogated in GPR109A knockout mice, confirming the receptor’s role in mediating anti-inflammatory responses in renal cells (69).

## 5 GPR109A in the retina

GPR109A is expressed in multiple tissues, including the kidneys, adipocytes, vascular endothelium, and immune cells. However, its role in the retina has attracted increasing attention due to its potential therapeutic relevance for retinal diseases such as DR. GPR109A is expressed in several key retinal cell types that contribute to barrier integrity and immune regulation. Additionally, several studies, including ours, have reported protective effects of various GPR109A agonists in different ocular ailments (Table 1).

**TABLE 1:** Relevance of GPR109A agonists in various retinopathies.

Agonist	Cell type	Treatment and dose	Findings	Validated through knockout models	References
Beta-hydroxybutyrate	Mouse retina	WT and GPR109A KO mice were injected intraperitoneally with BHB at 300 mg/kg once daily for 3 consecutive days, and on day 4 they received LPS at 4 mg/kg i.p. together with either PBS or BHB (300 mg/kg).	Systemic administration of BHB significantly attenuated LPS-induced ocular inflammation in wild-type mice, as evidenced by a marked reduction in retinal leukostasis and decreased infiltration of inflammatory myeloid cells in the eye.	Yes	(77)
Beta-hydroxybutyrate	RPE	In ARPE-19 and primary RPE cells, BHB was used at 5 mM, administered concurrently with TNF-α (10 ng/mL) to assess GPR109A-dependent suppression of inflammatory cytokine expression	BHB (5 mM) robustly suppressed TNF-α-induced IL-6 and Ccl2 expression in ARPE-19 and wild-type primary RPE cells, effects that were abolished in Gpr109a^/^ cells, demonstrating direct GPR109A-mediated anti-inflammatory signaling	Yes	(76)
Beta-hydroxybutyrate	Human REC Mouse retina	HRECs with GPR109A knockdown by siRNA were pretreated with 1, 3, or 5 mM BHB for 24 h and then challenged with VEGF 50 ng/ml WT and GPR109A KO mice challenged with LPS received 700 mg/kg BHB via i.p	BHB treatment resulted in significant increases in TEER, indicating improved barrier protection. Systemic βHB (700 mg/kg i.p) preserved blood-retinal barrier integrity and visual function by suppressing inflammation-induced vascular leakage through GPR109A signaling.	Yes	(79)
Beta-hydroxybutyrate	Mouse retina	Intraperitoneal (i.p) treatment twice weekly for 10-week period with 25, 50, or 100 mg/kg BHB in STZ induced diabetic mouse models	50 and 100 mg/kg BHB significantly reduced retinal ER stress markers (pPERK, pIRE1, ATF-6α), NLRP3 inflammasome activation markers (NLRP3, ASC, caspase-1) and inflammatory cytokines (IL-1β, IL-18).	No	(82)
Beta-hydroxybutyrate	Mouse retina	Intraperitoneal (i.p) treatment twice weekly for 10-week period with 25, 50, or 100 mg/kg BHB in STZ induced diabetic mouse models	50 and 100 mg/kg BHB significantly increased brain-derived neurotrophic factor (BDNF) and cell integrity (connexin 43) whilst autophagosome-lysosome formations (marked by LC3B and ATG14) significantly decreased.	No	(129)
Beta-hydroxybutyrate	Retinal ganglion cells (RGC)	βHB was co-injected intravitreally with N-methyl-D-aspartate (NMDA) at 50 nmol per eye.	Intravitreal βHB (50 nmol) delivered simultaneously with NMDA significantly protected retinal ganglion cells from NMDA-induced excitotoxic loss, reduced apoptosis, and dampened microglial activation in rat retina. These protective effects involved AMPK signaling rather than monocarboxylate transporter-mediated uptake.	No	(130)
Beta-hydroxybutyrate	Whole retina (photoreceptor layer, inner nuclear layer, ganglion cell layer)	Caloric restriction/intermittent fasting was used to elevate retinal 3HB in rats subjected to optic nerve and central retinal vessel transection (ONVT). Also, rats were subcutaneously injected with 3HB ∼ 1,000 mg/kg to mimic fasting-associated elevations	Repeated elevation of 3-hydroxybutyrate, achieved either by intermittent fasting or repeated subcutaneous administration, protected rat retina from acute ischemic degeneration in an ONVT model by preserving retinal structure and viability, preserving the pentose phosphate pathway, elevating the TCA intermediate fumarate to activate Nrf2-mediated antioxidant defenses, and enhancing ketolytic metabolic flux.	No	(131)
Beta-hydroxybutyrate	Mouse retina	Rd10 mice were subjected to ketogenic diet (KD) and ketogenic and low protein diet (KLP) to induce circulating BHB. Diets were given *ad libitum* from postnatal day (PD) 23 to PD50.	A ketogenic and low-protein diet slowed photoreceptor degeneration and preserved retinal structure and function in rd10 mice. Suggesting that elevation of BHB through ketosis plus low protein intake confers neuroprotection in this model of inherited retinal degeneration.	No	(132)
L-2-oxothiazolidine-4-carboxylic acid	RPE	ARPE-19 cells were pretreated with OTC at 0.25, 0.5, or 1.0 mM, followed by oxidative challenge with 300–500 μM H_2_O_2_ or inflammatory stimulation with TNF-α (10 ng/mL). In parallel, primary RPE cells isolated from wild-type and Gpr109a^/^ mice were stimulated with TNF-α (10 ng/mL) in the presence or absence of OTC to assess receptor involvement.	OTC attenuated oxidative stress and inflammatory responses in retinal pigment epithelial cells by increasing intracellular glutathione, reducing ROS accumulation, and suppressing pro-inflammatory cytokine expression. In primary RPE cells derived from wild-type and Gpr109a^/^ mice, OTC similarly reduced TNF-α–induced inflammatory signaling, indicating that while OTC can activate GPR109A, its protective effects are not entirely dependent on GPR109A signaling. These findings support OTC’s potential role as a redox-modulating therapeutic in retinal disease.	Yes	(122)
L-2-oxothiazolidine-4-carboxylate (OTC)	RPE	ARPE-19 and primary RPE cells were treated with OTC at concentrations ranging 0.25–1 mM in the presence of 500 μM H_2_O_2_ to induce oxidative stress.	OTC, a substrate for the sodium-coupled monocarboxylate transporter SLC5A8 (SMCT1), augmented intracellular glutathione and protected ARPE-19 and primary RPE cells from oxidative H_2_O_2_ injury in a transporter-dependent manner	No	(121)
Monomethyl-fumarate	Mouse retina (ganglion cell layer, Müller cell)	Mice received daily 100 mg/kg (i.p.) injections of MMF beginning after induction of retinal ischemia-reperfusion injury.	MMF promoted Nrf2-dependent neuroprotection, evidenced by reduced neuronal loss in the ganglion cell layer, suppressed inflammation and Müller gliosis, enhanced antioxidative gene expression, and improved ERG responses.	No	(133)
Monomethyl-fumarate	Mouse retina (photoreceptor layer and outer nuclear layer)	Mice received MMF by i.p injection at doses of 50 or 100 mg/kg, administered once prior to exposure to intense white light.	MMF treatment protected the retina from light-induced retinopathy, preserving outer nuclear layer thickness and photoreceptor integrity, maintaining ERG a- and b-wave responses, and suppressing microglial activation and pro-inflammatory gene expression; these protective effects were associated with modulation of GPR109A signaling and attenuation of oxidative and inflammatory stress in the neural retina.	No	(95)
Monomethyl-fumarate	Mouse retina	Mice were treated with dimethyl fumarate (DMF) administered intraperitoneally at doses of 15 mg/kg or 30 mg/kg body weight.	DMF significantly reduced light-induced retinal degeneration, increased retinal glutathione levels, and reduced microglial activation *in vivo*, although it did not show protection in the optic nerve crush model, demonstrating model-dependent neuroprotection measured by OCT and histology.	No	(134)
Monomethyl-fumarate	Mouse retina (retinal ganglion cells)	Mice received daily i.p injections of DMF at doses up to 100 mg/kg, administered following optic nerve crush.	Dimethyl fumarate significantly enhanced retinal ganglion cell survival and functional activity after optic nerve injury and upregulated Nrf2/HO-1 antioxidant signaling in the retina, indicating neuroprotective effects mediated through this pathway.	No	(135)
Dimethyl-fumarate	Mouse retina (retinal ganglion cells)	Mice received DMF by oral gavage at 30 mg/kg twice daily, administered either: preventively (starting the day after experimental autoimmune encephalomyelitis (EAE) induction), or interventionally (starting when motor deficits and visual acuity loss were already evident).	DMF treatment mitigated optic neuritis severity, preserved visual acuity and retinal ganglion cells, and reduced motor deficits in EAE mice, with greater efficacy when given after onset of symptoms, and a complementary clinical analysis showed increased time to optic neuritis recurrence in RRMS patients on DMF therapy.	No	(136)
Mono-ethyl-fumarate	RPE	ARPE-19 cells were treated with mono-ethyl fumarate (5–40 μM) prior to A2E and blue-light-induced oxidative injury	Mono-ethyl fumarate (MEF) dose-dependently protected RPE cells from oxidative injury, significantly increasing antioxidant defenses (HO-1, NQO1, SOD1), reducing intracellular ROS, and suppressing apoptosis, as evidenced by decreased Bax/Bcl-2 ratio and reduced caspase-3 activation.	No	(137)
	Mouse retina	In sodium iodate (SI)-induced retinal degeneration mouse model, animals were orally administered mono-ethyl fumarate (MEF) once daily at doses of 50, 100, or 200 mg/kg for 4 weeks, with a single sodium iodate (30 mg/kg i.p.) injection given at the beginning of week 2 to induce retinal degeneration.	Oral MEF treatment preserved retinal structure and thickness on OCT and histology, attenuated photoreceptor and outer retinal degeneration, upregulated antioxidant proteins (SOD1, GPX4) in retinal tissue, and reduced apoptosis markers, indicating robust anti-oxidative and anti-apoptotic retinal protection *in vivo*.		
Niacin	RPE	Primary RPE cells and ARPE-19 cells were exposed to TNF-α (10 ng/mL) for 24 h, with or without 1 mM niacin in the culture medium.	NA treatment (1 mM) induced GPR109A-dependent suppression of inflammatory cytokine expression by significantly reduced IL-6, Ccl2 expression.	Yes	(76)
Niacin	Human retina	Human clinical study of adults with central retinal vein occlusion (CRVO) were given high-dose niacin orally over 12 months.	Systemic oral niacin therapy in adults with central retinal vein occlusion showed a progressive improvement in visual acuity and a significant reduction in central macular thickness over 12 months compared with controls, suggesting potential functional and anatomic benefits in CRVO management.	No	(138)
Niacin	Choroidal circulation in fovea region	Human clinical study of patients with age-related macular degeneration (AMD) with bilateral drusen were given a single dose of oral niacin (250 mg or 500 mg).	Oral niacin (250–500 mg) caused a transient increase in choroidal blood volume in AMD patients at 30 min after ingestion, particularly at the 500 mg dose. This was accompanied by a decrease in choroidal blood velocity at the higher dose, such that overall flow did not change significantly. Suggesting that niacin modifies choroidal hemodynamics in AMD.	No	(139)
Niacin	Human retina	Patients were treated with oral nicotinic acid starting at 300 mg/day, increased by 300 mg each week to a maximum of 3,000 mg/day by week 10, maintained for two weeks, and then discontinued at week 12, with aspirin co-administered initially to reduce flushing.	High-dose oral nicotinic acid (titrated up to 3,000 mg/day over 10 weeks) in CRVO/BRVO patients was associated with improvements in best-corrected visual acuity, reductions in hemorrhages and edema, and improved visual field indices, along with beneficial changes in lipid profiles; treatment was generally tolerated though some systemic and ocular complications occurred.	No	(140)
Sodium butyrate	Whole mouse retina	Butyrate oral gavage at 200 or 500 mg/kg daily in oxygen induced retinopathy model of neonatal mice for ten days	Significant reduction in retinal vaso-obliteration and neovascularization in the 500 mg/kg NaB treatment. Smaller, potentially non-significant reduction in 200 mg/kg group.	No	(83)
	Human retinal endothelial cells	HRECs exposed to 12 h 2% oxygen, and treated with 1-, 2-, or 5-mM butyrate	Dose dependent inhibition of tube formation in cells treated with butyrate.		
Sodium butyrate	Mouse retina	STZ-induced diabetic mice received sodium butyrate at 200 mg/kg body weight per day by oral gavage for 12 weeks.	Sodium butyrate supplementation significantly ameliorated Type-1 diabetes induced DR in mice by lowering blood glucose, preserving retinal thickness and visual function, strengthening intestinal tight junctions, and modulating gut microbiota and plasma SCFA profiles, supporting a therapeutic role for butyrate via a gut-retina axis	No	(141)
Sodium butyrate	Mouse retina	Mice received sodium butyrate supplementation in drinking water at 5 g/L continuously for 12 weeks following HFD/STZ induction.	Butyrate supplementation mitigated early diabetic NVU impairment by lowering body weight and glucose, improving serum lipid profiles and hepatic steatosis, partially restoring gut microbial balance, and ameliorating retinal vascular and structural changes while enhancing visual behavior	No	(142)
Sodium butyrate	HUVECs	HUVECs were treated with sodium butyrate (NaBu) at 1, 2.5, or 5 mM to evaluate dose-dependent effects on endothelial proliferation and tube formation.	In cultured HUVECs, sodium butyrate dose-dependently inhibited endothelial proliferation and capillary-like tube formation, accompanied by upregulation of TXNIP and suppression of VEGFR2 signaling; genetic modulation of TXNIP (overexpression or knockdown), respectively, enhanced or reversed these anti-angiogenic effects, establishing TXNIP as a key mediator of butyrate action at the cellular level.	No	(143)
	Mouse choroid	Choroid explants were cultured with NaBu at 0.1, 0.5, 2.5, or 5 mM to assess sprouting inhibition.	In mouse choroid explant sprouting assays, sodium butyrate significantly and dose-dependently suppressed choroidal microvascular outgrowth, indicating a direct inhibitory effect on angiogenic sprouting from choroidal tissue independent of systemic influences.		
	Mouse retina	Mice received intravitreal injections of NaBu (1, 2.5, or 5 mM) on day 0 and day 3 post-laser photocoagulation, and CNV was assessed one week later	In a laser-induced choroidal neovascularization mouse model, intravitreal sodium butyrate administration markedly reduced CNV lesion size, demonstrating potent inhibition of pathological neovascularization *in vivo* and corroborating the anti-angiogenic effects observed *in vitro* and *ex vivo*.		
Sodium butyrate	Retinal astrocytes	Retinal astrocytes were pretreated with short-chain fatty acids such as acetate (C2, 10 mM), propionate (C3, 1 mM), or butyrate (C4, 1 mM) added 30 min prior to LPS exposure to assess modulation of inflammatory responses	In cultured retinal astrocytes, SCFAs (acetate, propionate, and butyrate) suppressed LPS-triggered production of pro-inflammatory cytokines (e.g., IL-6, TNF-α) and chemokines (e.g., CXCL1, CXCL12); however, under certain conditions SCFAs also enhanced astrocyte ability to activate T cells, indicating complex modulatory effects on innate and adaptive inflammation in astrocytes.	No	(144)
	Mouse retina	Mice received i.p. injection of butyrate (C4) at 500 mg/kg in 100 μL water, followed 30 min later by intravitreal LPS challenge to induce uveitis.	Systemic SCFA administration (notably butyrate at 500 mg/kg i.p.) reduced the severity of endotoxin-induced uveitis, evidenced by lower clinical inflammation scores, reduced leukocyte infiltration into the eye, and decreased retinal expression of inflammatory mediators compared with vehicle-treated controls, supporting a protective, anti-inflammatory effect of SCFAs in acute ocular inflammation.		

### 5.1 Retinal pigmented epithelial cells (RPE)

RPE cells form the outermost layer of the retina, adjacent to the choroid, a highly vascular structure that delivers oxygen and nutrients to the eye (70). These cells maintain the outer blood-retina barrier (oBRB) via tight junctions, regulating the entry of substances from systemic circulation. Disruption of RPE integrity leads to fluid influx and diabetic macular edema (DME), a major contributor to vision loss in DR (71, 72). Due to the retina’s high metabolic activity and constant exposure to photooxidation, RPE cells are equipped with melanin and antioxidant enzymes to mitigate ROS accumulation (73).

In 2009, a study from our laboratory confirmed GPR109A expression in mouse and human-derived RPE cells via real-time PCR and fluorescence *in situ* hybridization (FISH), with preferential localization to the basolateral membrane (74). This positioning allows GPR109A to interact with circulating endogenous and pharmacologic ligands. RPE cells secrete pro-inflammatory cytokines such as IL-6 and MCP-1 (75). Another study from our laboratory showed that TNFα-induced expression of these cytokines was attenuated by niacin and BHB, both of which are GPR109A agonists. Pertussis toxin (PTX), a Gi inhibitor, abolished these effects, confirming GPR109A’s role in mediating anti-inflammatory responses (76).

Emerging evidence strongly associates GPR109A with retinal pigment epithelium (RPE) homeostasis and suggests an important role in age-related macular degeneration (AMD). The bioRxiv study by Powell et al. (77) showed strong expression of GPR109A in RPE cells, where the cell lineage regulates retinal immune balance by decreasing the secretion of pro-inflammatory cytokines and reducing oxidative stress responses within the retinal environment. Loss-of-function studies in GPR109A-knockout mice showed that there is a gradual progressive loss of retinal morphology and function, including basement membrane thickening, increased oxidative stress, impaired barrier integrity, and accelerated neurodegeneration, all of which are in accordance with early AMD pathology. They reveal that GPR109A signaling regulates both RPE structural stability and prevents chronic inflammation–the primary drivers of AMD progression. Further analyses from the preprint support the notion that activation of GPR109A enhances an immunoinhibitory retinal milieu by modulating RPE–microglial crosstalk and attenuating peripheral immune cell infiltration (77). Taken together, these data suggest that the GPR109A pathway is a promising therapeutic target for AMD, and that activating it might slow or arrest RPE dysfunction, oxidative injury, and inflammation-related retinal degeneration.

### 5.2 Retinal endothelial cells (REC)

REC lines the retinal vasculature and forms the inner blood-retina barrier (iBRB), protecting the retina from toxins and inflammatory cells (78). A study from our laboratory reported GPR109A expression in human retinal endothelial cells, with localization to cell junctions (79). Furthermore, siRNA-mediated knockdown of GPR109A in HRECs decreased transepithelial electrical resistance (TEER), indicating compromised barrier function. BHB treatment to HRECs resulted in dose-dependent increases in TEER, suggesting that GPR109A activation enhances endothelial barrier integrity. Since BHB is a ketone body elevated during fatty acid oxidation, its therapeutic concentrations can be achieved via intermittent fasting or supplementation (80, 81). This is particularly relevant in diabetes, where elevated ketones are common.

Trotta et al. (82) study explores the protective role of hydroxycarboxylic acid receptor 2 (HCAR2) in diabetic retinal damage. Using a streptozotocin-induced diabetic mouse model, the researchers demonstrated that systemic administration of BHB, an endogenous HCAR2 ligand, significantly reduced retinal endoplasmic reticulum (ER) stress and NLRP3 inflammasome activation, both of which are key contributors to diabetic retinopathy. Treatment with BHB lowered levels of ER stress markers (pPERK, pIRE1, ATF-6α), inflammasome components (NLRP3, ASC, caspase-1), and pro-inflammatory cytokines (IL-1β, IL-18), while also decreasing retinal apoptosis and improving connexin 43 expression (82). These findings suggest that activating retinal HCAR2 receptors via systemic BHB may offer a novel anti-inflammatory and neuroprotective strategy against diabetic retinal damage.

Results from our bioRxiv preprint of sodium butyrate treatment with preclinical ROP models demonstrated that butyrate conferred a strong protective effect on retinal endothelial cells (83). Oral sodium butyrate daily significantly reduced pathological angiogenesis in an oxygen-induced retinopathy (OIR) model, suggesting that butyrate can modulate endothelial responses during the critical phases of retinal neovascularization. It is further reported in the preprint that treatment with butyrate also constrains dysregulated vascular growth while maintaining a more physiologic vascular architecture in the premature retina (83) These findings indicate that butyrate may stabilize endothelial responses to hypoxic stress and thereby counter the excessive neovascular drive observed in ROP. These endothelial-specific benefits were importantly described in wild-type mice; the study included no GPR109A knockouts (KOs). Therefore, it remains unexplored how much of butyrate’s endothelial action is mediated by GPR109A-dependent versus GPR109A-independent pathways.

### 5.3 Microglia

Microglia are the resident immune cells of the retina, primarily located near synapses in the outer plexiform layer (OPL) (84). They monitor the retinal environment and respond to injury or infection by releasing cytokines, activating the complement system, and phagocytosing debris (85). In DR, microglia are activated by hyperglycemia, ischemia, and inflammatory cytokines (86), as shown in both human and mouse models (87–89). Initially, microglia release anti-inflammatory cytokines (90), but prolonged insult leads to overproduction of pro-inflammatory mediators such as TNFα and IL-1β, which damage epithelial and endothelial junctions and increase vascular permeability (91–93). Their migration from the inner to outer retina exacerbates BRB breakdown and disease progression (94).

Jiang et al. (95) demonstrated that GPR109A expression increases in response to photooxidative stress. Depletion of microglia reduced this upregulation, confirming that retinal microglia express GPR109A (95). MMF, a GPR109A agonist, suppressed microglial activation and pro-inflammatory gene expression, favoring an anti-inflammatory state. These findings suggest that early GPR109A activation may delay DR onset by modulating microglial behavior.

Further, we showed (a preprint article) that GPR109A knockout mice had increased leukocyte infiltration and immune activation compared to wild-type controls (77). Following lipopolysaccharide (LPS) challenge, BHB treatment reduced leukocyte counts in wild-type but not GPR109A-deficient mice. Flow cytometry confirmed elevated CD45^+^ immune cells in knockout retinas, implicating GPR109A in suppressing inflammatory invasion, likely via modulation of microglia response. Importantly, GPR109A agonists like BHB offer a non-invasive alternative to intravitreal injections, which are associated with risks and are only applicable in advanced disease stages. These findings support the therapeutic potential of GPR109A activation in the management of early-stage retinopathies.

Our bioRxiv preprint also highlights a notable influence of butyrate on retinal microglial activation, a key contributor to neurovascular injury in ROP. Oral butyrate supplementation protected against microglia-associated inner retinal pathology in OIR mice, with reductions in inflammatory activation and improvements in overall retinal cellular health. The preprint further reports that butyrate’s protective effects extend across multiple retinal cell types, including microglia, especially during early-phase disease progression in both standard OIR and hyperglycemia-associated retinopathy models (83). These findings suggest that butyrate may help modulate microglial inflammatory responses that otherwise exacerbate vascular and neuronal damage in premature retinas. However, similar to the endothelial findings, the authors did not validate these microglial mechanisms using GPR109A-KO mice, leaving the specific receptor contribution unresolved and highlighting a key next step for mechanistic clarification.

## 6 Therapeutic agonists and drug development

To introduce the therapeutic relevance of GPR109A agonists in retinal disease, this section reviews considerations for biased agonism, selection of delivery routes (oral, topical and intravitreal), and emerging combinatorial approaches with anti-VEGF therapy.

### 6.1 Niacin, BHB, butyrate: efficacy and limitations

Nicotinic acid (niacin), a vitamin of the B complex, was the first agonist discovered for GPR109A (26, 27), earning it the name “niacin receptor 1.” Other endogenous agonists include BHB and butyrate (29, 96, 97). Niacin is considered the most potent, with EC50 values of ∼3 μM for mouse and ∼1 μM for human receptors (27), although endogenous levels are insufficient to activate the receptor.

BHB has been shown to have higher potency than butyrate (29), with an EC50 of ∼750 μM (98). Basal BHB levels (50–400 μM) are below the activation threshold but can rise to 1–2 mM during short-term starvation (99, 100), 6–8 mM with prolonged fasting (101), and 1–2 mM after intense exercise (102). Nutritional ketosis can elevate BHB to ∼5 mM (103). Butyrate, produced by bacterial fermentation of dietary fiber, activates GPR109A with an EC50 of ∼1.6 mM. Despite high colonic concentrations (∼20 mM), circulating butyrate (∼5 μM) is insufficient for activation.

Niacin, BHB, and butyrate are not exclusive to GPR109A. Niacin also weakly binds to GPR109B and is an NAD^+^ precursor (104). Butyrate and BHB activate GPR41 and GPR43 (54, 105, 106), and are transported via monocarboxylate transporters (107). They inhibit histone deacetylases (108), increase intracellular Ca^2+^ (109), and enhance mitochondrial ATP production (110).

Although niacin remains the most potent classical GPR109A agonist, its clinical use is limited by dose-dependent flushing mediated through β-arrestin-induced PGD_2_ release. This adverse effect underscores the therapeutic value of biased agonism-developing ligands that preferentially activate beneficial anti-inflammatory or antioxidant pathways while minimizing β–arrestin–driven flushing. Such strategies may allow more selective engagement of GPR109A signaling for retinal indications.

### 6.2 Synthetic agonists: MMF, MK6892, GSK256073

Synthetic agonists have been developed to better understand GPR109A signaling and to explore therapeutic applications. These include niacin derivatives such as acipimox and acifran, which have high affinity for GPR109A and low affinity for GPR109B (111, 112). MMF, the active metabolite of dimethyl fumarate (DMF), is used in the treatment of psoriasis and multiple sclerosis and also activates GPR109A (113, 114).

MMF mitigates microglial activation and upregulates antioxidant genes such as HMOX1 while suppressing pro-inflammatory genes, including NLRP3, Caspase-1, IL-1β, and TNF-α, in light-induced retinopathy models (95). These mechanisms are relevant to DR, which also involves upregulation of inflammatory genes (115, 116). MMF’s ability to enhance heme oxygenase 1 (HMOX1) expression may help regulate oxidative stress in DR (95, 117).

Other synthetic agonists include MK6892 and GSK256073, which have high affinity for GPR109A and reduced flushing profiles (118, 119). MK-1903 was developed for dyslipidemia (120), while MK0354 retained antilipolytic effects with reduced flushing (40). GSK256073 has shown glucose-lowering effects in patients with type 2 diabetes (118). Compared to niacin, these newer agonists offer potential advantages, including reduced flushing, greater pathway selectivity, and possible biased agonism that favors anti-inflammatory and antioxidant signaling. Different pharmacologic profiles offer options for differential delivery strategies, such as oral use (MMF) and potential topical or intravitreal formulations under development to optimize retinal bioavailability. However, none of these agents has yet received clinical approval for the treatment of retinal diseases.

Since VEGF is central to retinal neovascular diseases, combining GPR109A agonists with anti-VEGF agents is an emerging therapeutic concept. Agonists like MMF and MK6892, which suppress inflammatory and oxidative pathways upstream of VEGF induction, may complement standard intravitreal anti-VEGF therapies by improving durability, reducing injection burden, and addressing inflammation-driven components of disease that VEGF-neutralization alone does not target.

### 6.3 OTC: dual antioxidant and anti-inflammatory effects

OTC, a cysteine prodrug, has emerged as a promising GPR109A agonist with antioxidant and anti-inflammatory properties. OTC is transported into cells via the sodium-dependent monocarboxylate transporter SLC5A8, where it enhances glutathione production in RPE cells (121). In TNFα-treated RPE cells, OTC significantly reduced IL-6 and MCP-1 secretion compared to niacin. Similar effects were observed *in vivo*, with reduced IL-1β levels in mouse retinas.

Interestingly, OTC’s anti-inflammatory effects were independent of SLC5A8. In SLC5A8 knockout RPE cells, OTC still suppressed TNFα-induced IL-6, suggesting direct activation (122). Although OTC has a lower affinity for GPR109A than niacin, it may be more effective at reducing inflammation and oxidative stress. Inflammation and oxidative stress are interdependent; each exacerbates the other (123, 124). IL-6 and other cytokines increase oxidative stress by altering NADPH oxidase expression (125). Several studies have highlighted the protective role of various GPR109A agonists in multiple retinopathies; however, it is important to note that although these studies use GPR109A agonists, some have not thoroughly investigated whether the observed effects are exclusively mediated through GPR109A signaling, as certain agonists are known to exert receptor-independent effects (Table 1).

Niacin, MMF, MK6892, GSK256073, and OTC represent distinct classes of GPR109A agonists with varying potencies, pathway preferences, and delivery potentials. Niacin is potent but limited by flushing, whereas MMF and MK6892 may have more favorable biased signaling profiles. OTC also provides dual antioxidant and anti-inflammatory actions that complement GPR109A signaling. Gaining insight into these differences will be critical when designing non-invasive delivery routes, such as oral or topical formulations, or in future combination approaches employing anti-VEGF agents for retinal disease.

## 7 Challenges and future directions

### 7.1 Cutaneous flushing and off-target effects

One of the most significant challenges in the clinical use of GPR109A agonists is cutaneous flushing, particularly associated with niacin. This reaction is mediated by GPR109A activation in dermal Langerhans cells, leading to prostaglandin D2 release and vasodilation, which causes flushing and burning pruritus (40, 126). Additionally, niacin has been shown to increase blood glucose and HbA1c levels (127), limiting its utility in diabetic patients. Rare but serious ocular side effects, such as cystoid macular edema (CME), have also been reported (128). These off-target effects underscore the need for more selective and better-tolerated GPR109A agonists.

### 7.2 Potential for early-stage DR intervention

Current treatments for DR are largely reactive, initiated only after significant retinal damage has occurred. GPR109A agonists, particularly those with anti-inflammatory and antioxidant properties, offer a promising opportunity for early-stage intervention. By preserving the blood-retina barrier and modulating immune responses, GPR109A activation may delay or prevent the onset of clinically detectable DR. This preventive approach could significantly improve long-term visual outcomes and reduce the burden of invasive procedures such as laser therapy and intravitreal injections.

### 7.3 Clinical trial prospects and translational hurdles

Despite compelling preclinical data, clinical translation of GPR109A-targeted therapies faces several hurdles. These include the need for well-characterized, non-flushing agonists, retina-specific pharmacokinetics, and validated biomarkers of GPR109A activation in ocular tissues. Major translational challenges are efficient drug delivery to posterior segment tissues (oral, topical, and intravitreal routes each present specific challenges), addressing systemic side effects (e.g., niacin-associated flushing), and addressing patient-to-patient heterogeneity in receptor expression and pharmacodynamics. Regulatory approval will also require robust safety and efficacy data from human trials. Ongoing early-phase trials should include response-predictive biomarkers, pragmatic add-on designs with anti-VEGF therapy, and endpoints that capture anti-inflammatory and barrier-protective benefits beyond visual acuity. Currently, MMF is the only GPR109A agonist with demonstrated efficacy in retinal disease models, but no commercial drugs targeting GPR109A have yet been approved for ocular indications. Future approaches would focus on personalized patient selection, designing long-acting and non-invasive formulations, and rational combination treatment regimens including anti-VEGF agents to enhance durability and address pathogenic pathways not targeted by VEGF neutralization.

## 8 Conclusion

GPR109A has emerged as a compelling therapeutic target in retinal diseases, particularly DR. Its activation by endogenous and synthetic agonists has demonstrated potent anti-inflammatory, antioxidant, and barrier-protective effects across key retinal cell types, including retinal pigment epithelial cells, endothelial cells, and microglia. These mechanisms not only address the core pathophysiological drivers of DR (inflammation and oxidative stress) but also provide a pathway to preserve retinal integrity before irreversible damage occurs. The receptor’s ability to modulate immune responses and maintain vascular homeostasis positions it as a promising candidate for early-stage, non-invasive intervention strategies.

Despite encouraging preclinical data, translating GPR109A-targeted therapies into clinical practice requires further investigation. Challenges such as cutaneous flushing, off-target effects, and the need for retina-specific delivery systems must be addressed to ensure safety and efficacy. Moreover, the development of biased agonists and novel compounds, such as OTC and MMF, highlights the potential to design targeted therapies with improved tolerability. Future research should focus on validating these findings in clinical trials, optimizing delivery platforms, and identifying biomarkers of GPR109A activation in ocular tissues. Continued exploration of GPR109A signaling may ultimately lead to a paradigm shift in the prevention and management of retinal diseases.
